# Vonoprazan as an adjuvant to cisplatin: enhancing antitumor efficacy while mitigating nephrotoxicity and gastrointestinal adverse effects

**DOI:** 10.3389/fphar.2026.1798103

**Published:** 2026-04-30

**Authors:** Faiz N. Alenezi, Marwa E. Abdelmageed, Manar A. Nader, Marwa S. Zaghloul

**Affiliations:** 1 Department of Pharmacology and Toxicology, Faculty of Pharmacy, Mansoura University, Mansoura, Egypt; 2 Department of Pharmacology and Toxicology, Faculty of Pharmacy, Mansoura National University, Gamasa, Egypt

**Keywords:** cisplatin, vonoprazan, inflammasome, 3-(4,5-dimethylthiazol-2- yl)-2,5-diphenyltetrazolium bromide, autophagy, oxidative stress, apoptosis, synergistic anticancer effect

## Abstract

**Background/Objective:**

Cisplatin is a popular platinum-containing chemotherapeutic agent that has been used clinically since the 1970s to manage a wide range of hematologic and solid tumors. However, its therapeutic purpose is restricted by significant dose-related toxicities, particularly nephrotoxicity and gastrointestinal damage. Therefore, the present study assesses the potential protective effects of vonoprazan (a potassium-competitive acid blocker) against cisplatin-provoked organ damage, in addition to examining the impacts of vonoprazan on the antineoplastic activity of cisplatin.

**Methods:**

Rats were pretreated with vonoprazan (10 or 20 mg/kg) in an *in vivo* study, and their renal, gastric, and intestinal injuries were assessed using spectrophotometric assays, enzyme-linked immunosorbent assay (ELISA), and immunohistochemical (IHC) analysis. Moreover, the oxidative stress, inflammatory, autophagic, and apoptotic pathways were evaluated. Then, MCF-7 human breast cancer cells were treated with cisplatin alone or in combination with vonoprazan in an *in vitro* study, and the cell viability, combination index, and apoptosis-related markers were analyzed.

**Results:**

The *in vivo* study showed that vonoprazan pre-administration meaningfully lowered the injurious influences of cisplatin on the kidney, stomach, and intestine, as evidenced by improvements in the histopathological changes and decreased levels of serum lactate dehydrogenase (LDH), serum creatinine (sCr), and blood urea nitrogen (BUN). Vonoprazan boosted the antioxidant defenses by increasing the total antioxidant capacity (TAC) and decreasing the malondialdehyde (MDA) levels in the kidney, stomach, and intestinal tissues. Moreover, vonoprazan decreased the early biomarkers of acute kidney injury (KIM-1 and NGAL), improved the gastroprotective mediators (increased cGMP and PGI2; decreased serotonin), and modulated the intestinal epithelial and mucosal injury markers (increased TFF3; decreased IFABP). Vonoprazan also attenuated the inflammasome component (NLRP3) and inflammatory signaling mediators (NF-κB and IL-6) while modulating the autophagy-lysosomal pathway (enhanced LC3 and Beclin-1; decreased p62). However, the *in vitro* study results revealed that the combination of cisplatin and vonoprazan had a synergistic cytotoxic effect and an enhancing effect on the apoptotic pathway (increased p53 and BAX; decreased BCL2).

**Conclusion:**

Vonoprazan attenuates cisplatin-induced organ injury while augmenting its anticancer effects; this suggests the potential of vonoprazan as a supportive therapeutic strategy during cisplatin-based chemotherapy.

## 1 Introduction

Cisplatin is an anticancer drug that was first recognized in the 1970s; since then, it has been used to treat several hematologic and solid malignant tumors, including testicular, bladder, breast, cervical, and endometrial carcinomas (Ozols et al., 2003), along with gestational trophoblastic neoplasia as an adjuvant therapy (Gold and Raja, 2023). Cisplatin or cis-diamminedichloroplatinum (II) is a type of metallic platinum compound characterized by a square planar geometry that promotes DNA crosslinking, thereby blocking DNA replication and transcription to cause cell death (Gold and Raja, 2023; Ozols et al., 2003). Despite its therapeutic value as a cornerstone in managing a broad spectrum of malignancies, the use of cisplatin is often restricted by serious dose-dependent toxicities, notably nephrotoxicity and gastrointestinal (GI) injury. Extensive evidence has confirmed the devastating impacts of cisplatin on bodily organs, including the kidneys and stomach to a lesser extent (Cabezos et al., 2010; Perše and Večerić-Haler, 2018; Sharma and Gupta, 1997). Considering the crucial role of cisplatin in cancer therapy and its considerable side effects and toxicity that limit clinical use, it has become necessary to investigate and develop novel approaches that can reduce the toxic effects without compromising the therapeutic anticancer efficacy.

Vonoprazan is a potassium-competitive acid blocker (P-CAB) that has been used as a remedy for abdominal ulcers, erosive esophagitis, and *Helicobacter pylori* infection, as well as preventing ulcers induced by non-steroidal anti-inflammatory drugs (NSAIDs) or low-dose aspirin (Sugano, 2018). Vonoprazan reversibly attaches to the potassium-binding site of the H^+^/K^+^-ATPase enzyme, causing fast and continuous inhibition of gastric acid secretion (Padwale et al., 2024). Despite the increasing clinical use of vonoprazan as a potent P-CAB, research on its protective or beneficial effects on target organ damage, particularly the kidneys, liver, and intestines, remain minimal. Most available studies have focused primarily on its acid suppression efficacy and GI healing ability, but there are very few investigations of its antioxidant, anti-inflammatory, or cytoprotective roles in extragastric organs. Experimental evidence supporting the potential antioxidative actions of vonoprazan are limited to a few animal studies, such as the rat giardiasis model in which co-administration of vonoprazan and metronidazole reduced oxidative stress, suggesting indirect tissue protection rather than direct organ-specific effects (Sallam et al., 2025). Furthermore, current human and preclinical literature lack detailed biochemical or histopathological evaluations demonstrating the manner in which vonoprazan ameliorates the impacts from renal, hepatic, or intestinal oxidative or inflammatory injury. Consequently, evidence supporting any encouraging or protective effects of vonoprazan on target-organ damage is minimal and inconclusive, warranting future mechanistic and translational investigations.

Although the protective effects of vonoprazan in preventing stomach ulcers are well established in clinical trials and *in vitro* studies (Padwale et al., 2024; Sugano, 2018), very little is known about its efficacy in ameliorating cisplatin toxicity on other body organs, such as the kidneys. Accordingly, the present study intended to assess the potential protective actions of vonoprazan against cisplatin-induced organ damage, in addition to assessing different biochemical and molecular changes involved in the protective and cytotoxic effects afforded by vonoprazan. Additionally, we examined the impacts of vonoprazan on the antineoplastic activity of cisplatin. The overall objective of this study was to determine whether concurrent administration of vonoprazan causes synergistic, antagonistic, or no effects on cisplatin-induced cytotoxicity in cancer cells.

## 2 Materials and methods

### 2.1 Materials

Cisplatin was obtained as injectable vials (Cisplatine® Mylan (1 mg/mL), Oncotec Pharma Production, Dessau-Rosslau, Germany), and vonoprazan was obtained from Inspire Pharmaceutical Company (Cairo, Egypt). All supplementary chemicals and reagents used in the experiments were of analytical-grade purity.

### 2.2 *In vivo* study

#### 2.2.1 Experimental animals

Thirty-five male Wistar rats (200–250 g) were sourced from the Urology and Nephrology Centre at Mansoura University in Egypt and maintained in discrete areas in a biologically restrained area at constant temperature (25 °C ± 2 °C) and humidity (75% ± 5%) with 12-h light/dark cycles. The investigational procedures used in this work were in accordance with the principles and strategies for maintenance and management of investigational animals, as established by the Research Ethics Committee of the Faculty of Pharmacy at Mansoura University (approval code no. MU-ACUC, PHARM. PhD. 23.08.27), and adhered to the “Principles of Laboratory Animal Care” (NIH publication no. 85–23, revised 1985).

Animal welfare was carefully monitored daily throughout the experimental period to prevent potential pain or distress. The rats were observed daily for indicators of discomfort or distress, including variations in the general appearance, behaviors, bodyweights, and food and water intake amounts. The clinical signs, including respiratory rate and mobility, were also assessed. All monitoring procedures were handled by trained personnel experienced in recognizing normal species-specific behaviors and physical characteristics. The animals were excluded from data analyses if they developed signs of unexpected illness, such as infection, or if they did not receive the complete dose of cisplatin or vonoprazan owing to technical issues related to drug administration, anesthesia, or sample collection.

#### 2.2.2 Experimental design

The rats were arbitrarily distributed into five groups (7 rats/group) as follows. In the control group, the rats were administered 0.5% (w/v) CMC-Na solution orally for 10 consecutive days, along with a single intraperitoneal (i.p.) injection of normal saline on the seventh day of the experiment. In the vonoprazan-only control group, the animals received vonoprazan suspended in 0.5% (w/v) CMC-Na solution at an oral dose of 20 mg/kg for 10 d and were given i.p. normal saline on the seventh day of the study. In the cisplatin group, the rats were administered CMC-Na solution orally for 10 consecutive days, along with a single i.p. injection of cisplatin (8 mg/kg) on the seventh day of the experiment (Gong et al., 2021; Kumaş-Kulualp et al., 2023; Malik et al., 2015; Sahu et al., 2019). In the cisplatin + vonoprazan (10 mg/kg) group, the rats were treated with vonoprazan (10 mg/kg) for 10 consecutive days; on the seventh day of the experiment, they additionally received a single i.p. injection of cisplatin (8 mg/kg). In the cisplatin + vonoprazan (20 mg/kg) group, the rats were administered vonoprazan (20 mg/kg) daily for 10 d, along with a single i.p. administration of cisplatin (8 mg/kg) on the seventh day of the experiment. The treatment regimen and selected dosages were determined via a pilot study to evaluate the preliminary effects of cisplatin on the kidneys, stomach, and duodenum. The vonoprazan doses were selected through a preliminary pilot study in light of previously published experimental reports using similar dose ranges (Zhou et al., 2023; Chen et al., 2020; Sallam et al., 2025) to ensure optimal efficacy with acceptable safety in our model.

Upon completion of the study, on the eleventh day, the rats were anesthetized using thiopental sodium (50 mg/kg, i.p.) and weighed before calculating the percentage change in bodyweight. Then, blood samples were obtained via retro-orbital puncture, and the samples were allowed to settle for 30 min before centrifugation (1,000* g*, 15 min, 4 °C) to extract the serum. The collected sera were stored at −80 °C and used to evaluate various biochemical parameters. Next, the abdomen of each animal was opened to harvest the kidneys, stomach, and duodenum; these organs were then cleaned, perfused with ice-cold saline, and weighed before determining the kidney/bodyweight ratio. The left kidney as well as parts of the stomach and duodenum of each animal were promptly maintained in 10% neutral-buffered formalin for histological assessment and immunohistochemical (IHC) analysis, while the right kidney and remaining parts of the stomach and duodenum were weighed to prepare a 10% (w/v) homogenate. The collected organs were homogenized in ice-cold phosphate buffer (0.01 M, pH 7.4) and centrifuged at 3,000 rpm and 4 °C for 20 min. The supernatants were maintained at −80 °C to assess the oxidative stress values and perform the ELISA assessments. Finally, we followed a two-step euthanasia process for the rats, where we injected thiopental sodium for serum and tissue assembly in the initial anesthesia step and then performed exsanguination via cardiac puncture.

#### 2.2.3 Assessments

##### 2.2.3.1 Evaluation of kidney function biochemical markers

Commercially available kits were used to assess the serum creatinine (sCr) (cat. no. MD1001111, Spinreact, Girona, Spain), blood urea nitrogen (BUN) (cat. no. TK41041, Spinreact, Girona, Spain), and lactate dehydrogenase (LDH) (cat. no. 11407001, Agappe, Kerala, India) levels according to their manufacturers’ procedures.

##### 2.2.3.2 Assessment of oxidant/antioxidant levels

The renal, gastric, and duodenal malondialdehyde (MDA) and total antioxidant capacity (TAC) levels were assessed using commercial kits (Biodiagnostic kits, Cairo, Egypt; cat. nos. MD2529 and TA2513, respectively) as per manufacturer instructions.

##### 2.2.3.3 Enzyme-linked immunosorbent assay

Commercially available kits were used to assess the levels of kidney injury molecule-1 (KIM-1; cat. no. CSB-E08808r, Cusabio, TX, United States), neutrophil gelatinase-associated lipocalin (NGAL; cat. no. CSB-E09409r, Cusabio), nuclear factor kappa B subunit (NF-κB; cat. no. CSB-E13148r, Cusabio), NOD-, LRR-, and pyrin domain-containing protein 3 (NLRP3; cat. no. ER1965, Finetest, Hubei, China), microtubule-associated protein 1A/1B-light chain 3 beta (LC3B; cat. no. EK721185, AFG Bioscience, Northbrook, IL, United States), coiled-coil moesin-like BCL2-interacting protein (Beclin-1; cat. no. CSB-EL002658RA, Cusabio), cyclic guanosine monophosphate (cGMP; cat. no. RTEB1737, Assay Genie, Dublin, Ireland), prostacyclin (PGI2; cat. no. CSB-E13706r, Cusabio), serotonin (5-HT; cat. no. RTEB1749, Assay Genie), intestinal fatty-acid-binding protein (IFABP; cat. no. MBS164325, MyBioSource, CA, United States), trefoil factor 3 (TFF3; cat. no. RTF21-K01, Eagle Biosciences, NH, United States) from tissue homogenates using ELISA technique according to manufacturer protocols.

##### 2.2.3.4 Histopathological examination

For the histological examinations, samples of the kidneys, stomach, duodenum, and jejunum were preserved in 10% neutral-buffered formalin before being embedded in paraffin using a tissue processor (ASP 300S, Leica Microsystem, Germany). Then, hematoxylin and eosin (H&E) staining was applied to the paraffin-embedded tissues after cutting into slices of thickness 3–4 μm using a microtome (RM 2265, Leica Microsystem, Germany). The prepared slides from each group were scrutinized under a light microscope (Olympus CH2, Japan). The histopathological examinations were performed on three non-consecutive sections per organ per rat (kidney, stomach, duodenum, and jejunum). From each section, four non-overlapping microscopic fields were randomly selected and examined; then, all selected tissues were semi-quantitatively scored according to Tables 1–3.

**Table 1:** Criteria for histopathologic scoring of rat kidney lesions.

Score	Tubular damage	Tubular dilation and cast	Inflammation	Fibrosis
0	None	None	None	None
1	Minimal and few	Minimal dilation with rare-to-few intraluminal casts	Few, rare	Few
2	Mild-to-moderate tubular degeneration	Minimal-to-mild dilation with moderate number of intraluminal casts	Mild, focal	Moderate interstitial fibrosis
3	Diffuse and many instances of tubular necrosis	Numerous intraluminal casts	Moderate-to-severe coalescing interstitial inflammatory aggregates	Severe and dense interstitial fibrosis

**Table 2:** Criteria for histopathologic scoring of rat intestine lesions.

Score	Mucosal and crypt degenerative changes	Inflammation
0 (none)	No pathological changes	Absent
1 (mild)	Mild-to-rare mucosal injury with slight crypt and epithelial damage (basal third damage)	Absent-to-minimal
2 (moderate)	Mucosal and submucosal injury with basal two-third damage of crypt	Scattered-to-multifocal
3 (severe)	Diffuse and severe transmural damage with complete loss of crypt and epithelium	Diffuse and widespread

**Table 3:** Criteria for histopathologic scoring of rat stomach lesion.

Score	Mucosal degenerative changes	Inflammation
0 (none)	No pathological changes	Absent
1 (mild)	Minimal gastric gland vacuolation or necrosis	Absent-to-minimal
2 (moderate)	Moderate glandular vacuolation	Scattered-to-multifocal
3 (severe)	Severe glandular vacuolation or necrosis	Diffuse and widespread

##### 2.2.3.5 IHC analysis

The expression levels of interleukin-6 (IL-6) and sequestosome 1 (SQSTM1/p62) in the kidneys, stomach, and duodenum were assessed immunohistochemically using the avidin-biotin complex (ABC) method (Guesdon et al., 1979) with polyclonal antibodies (Thermo Fisher Scientific Anatomical Pathology; cat. nos. PA5-27617 for IL-6 and PA5-20839 for p62). The immunostained sections were coded prior to inspection to ensure blinded evaluation under a light microscope (Olympus CH2, Japan), thereby preventing knowledge of the experimental groups. For each slide (representing one animal), four distinct sections were analyzed using ImageJ (FIJI 2.10.0, National Institutes of Health, MD, United States), and one photograph was captured per section (Gupta et al., 2003).

### 2.3 *In vitro* cytotoxicity assay

To examine the individual and combined effects of vonoprazan and cisplatin, the viability of human breast cancer (MCF-7) cells was measured using the 3-(4,5-dimethylthiazol-2-yl)-2,5-diphenyltetrazolium bromide (MTT) assay.

#### 2.3.1 Cell culture

Human breast cancer (MCF-7) cells were maintained in Dulbecco’s modified Eagle medium (DMEM) supplemented with 100 μg/mL of streptomycin, 100 U/mL of penicillin, and 10% heat-inactivated fetal bovine serum (FBS). The cultures were incubated at 37 °C in a humidified atmosphere containing 5% CO_2_.

#### 2.3.2 Cell viability assay

The MTT assay was used to assess cell viability (Gasparini et al., 2017). In brief, the cells were inoculated in a 96-well tissue culture plate at a density of 1 × 10^5^ cells/mL (100 μL/well) and incubated at 37 °C for 24 h to facilitate monolayer formation. Once confluence was achieved, the culture medium was discarded and cell layer was washed twice with a washing solution. Then, two-fold dilutions of the samples (with cisplatin and vonoprazan) were screened individually and in combination in a concentration-dependent manner. Serial dilutions were next prepared in RPMI medium supplemented with 2% serum (maintenance medium). Each dilution (0.1 mL) was dispensed into a separate well, and three wells filled with only the maintenance medium were separately designated as controls. The culture plate was then incubated at 37 °C before the evaluations. After 48 h of applying numerous concentrations of vonoprazan (10–100 µM), cisplatin (1–50 μM), and their combination to evaluate cytotoxicity, 20 μL of the MTT solution (5 mg/mL in PBS) was prepared and dispensed into each well; the plate was then agitated on a shaker at 150 rpm for 5 min to achieve uniform mixing of the MTT solution and again incubated at 37 °C with 5% CO_2_ for 4 h to allow MTT metabolism. After incubation, the medium was discarded, and the plate was dried on paper towels as needed. The resulting formazan crystals (MTT metabolic product) were dissolved in 200 μL of dimethyl sulfoxide (DMSO) and shaken (150 rpm for 5 min) to completely mix the formazan with the solvent. The optical density (OD) of the solution was measured at 560 nm, and the OD of the background was measured at 620 nm.

Next, to assess the IC_50_ values of cisplatin and vonoprazan, subcytotoxic concentrations (IC_10_ and IC_30_) were chosen for the subsequent combination experiments; the IC_10_ and IC_30_ concentrations are suggested in combination studies to lower the level of extreme individual-agent cytotoxicity and to permit accurate estimation of the pharmacological interactions between agents. This is because using combination agents at subinhibitory concentrations permits assessment of the synergistic, additive, or antagonistic effects in the absence of confounding results triggered by maximal cytotoxicity of the individual compounds (Chou, 2010). The concentrations of cisplatin and vonoprazan used in the combination index (CI) analysis were determined from preliminary dose–response cytotoxicity experiments. These doses were selected to cover a suitable spectrum of fractional effects (Fa) to ensure reliable CI estimation. Specifically, concentrations close to the IC_10_ and IC_30_ values were chosen to explore possible synergistic interactions while avoiding excess cytotoxicity from either drug alone. This strategy allows more precise evaluation of the drug–drug interactions in accordance with the Chou–Talalay method. Accordingly, the concentrations presented in Table 4 represent the graded subinhibitory levels around the IC_10_–IC_30_ range for each of the agents to enable accurate CI calculation. Thus, cisplatin and vonoprazan were combined at concentrations equivalent to their respective IC_10_ and IC_30_ values, and the cell viability was assessed using the MTT assay after 48 h. The inhibitory effects of vonoprazan and cisplatin alone and in combination were estimated via the CI (Chou, 2010) using the Chou–Talalay method (CompuSyn software, ComboSyn Inc., Paramus, NJ, United States). The CI serves as a metric for quantitative evaluation of the interactions of cisplatin and vonoprazan, where CI < 1 denotes a synergistic effect and CI > 1 denotes an antagonistic association (Chou, 2010; Chou and Talalay, 1984).

**TABLE 4:** Combination index (CI) values for different concentrations of cisplatin and vonoprazan against the human breast cancer cell line (MCF-7).

Cisplatin (µM)	Vonoprazan (µM)	CI
30	60	0.5
8.5	28.5	0.75
2.7	13.5	0.9
1.3	8	0.95
0.75	5.5	0.97

CI: combination index; CI < 1, synergistic effect; CI > 1, antagonistic association.

#### 2.3.3 Apoptosis assay

Commercially available tumor protein p53 (p53), B-cell lymphoma 2 (BCL2), and BCL2-associated X protein (BAX) assay kits were used for the apoptosis assay (cat. nos. HUDC0069, HUEB0345, and HUFI00482; Assay Genie, Dublin, Ireland). In brief, 1 × 10^6^ cells/mL were treated with cisplatin, vonoprazan, or their combination for 48 h; following incubation, the cell lysates were obtained using ice-cold cell lysis buffer as per manufacturer guidelines. A total of 100 µg of protein was adjusted to 50 µL and blended with 50 µL of 2X reaction buffer, which included 10 mM of dithiothreitol and 5 µL of the assay substrate. The samples were then incubated at 37 °C for 2 h before measuring the absorbance at 450 nm.

### 2.4 Statistical analysis

The data were statistically analyzed using the Statistical Package for the Social Sciences (SPSS 25.0, IBM/SPSS Inc., Chicago, IL, United States). The assumptions of normality for each group and homogeneity of variances were assessed using the Shapiro–Wilk and Levine’s tests, respectively. Then, one-way analysis of variance (ANOVA) followed by Tukey–Kramer *post hoc* test was used to assess the significant differences between more than two normally distributed groups of continuous data. The normally distributed data were presented as the mean ± standard deviation (SD), and a *p-*value < 0.05 was used as the threshold for statistical significance.

## 3 Results

### 3.1 *In vivo* study

#### 3.1.1 Influences of vonoprazan on cisplatin-mediated alterations in the percent change in bodyweight, kidney/bodyweight ratio, and serum kidney function biomarkers and LDH

**FIGURE 1:**
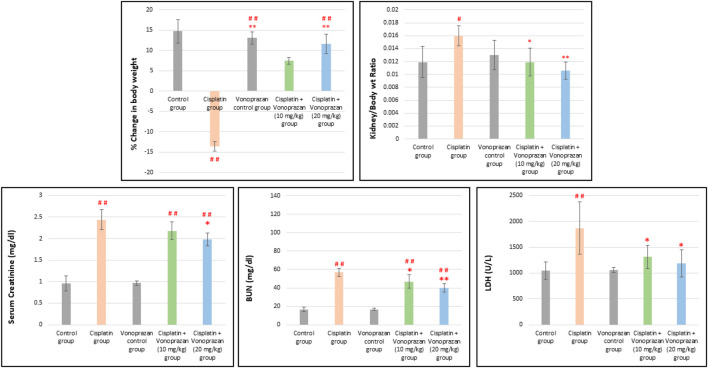
Influences of vonoprazan on cisplatin-mediated alterations in the percent change in bodyweight, kidney/bodyweight ratio, and serum kidney function biomarkers and LDH. The data are presented as the mean ± standard deviation (SD) (n = 7). Cisplatin was administered as a single dose (8 mg/kg, i.p.) on the seventh day, while vonoprazan was orally administered at two dose levels (10 or 20 mg/kg) for 10 consecutive days. Statistical evaluations were carried out using one-way ANOVA, followed by the Tukey–Kramer *post hoc* test; # denotes a significant difference over the control group, ## indicates a highly significant difference over the control group, * represents a significant difference over the cisplatin group, and ** indicates a highly significant difference over the cisplatin group. BUN, blood urea nitrogen; LDH, lactate dehydrogenase.

As observed from the results, cisplatin administration triggered a momentous diminution in total bodyweight compared to the control group while vonoprazan (10 and 20 mg/kg) restored the normal rate of increase in bodyweight. Additionally, cisplatin induced a meaningful increase in the kidney/bodyweight ratio compared to the control group; in contrast, the vonoprazan group showed a substantial decrease in kidney/bodyweight ratio compared to the cisplatin group. Cisplatin treatment triggered a noteworthy elevation in the serum levels of the kidney function (sCr and BUN) and tissue damage (LDH) biomarkers compared to the control group. In contrast, coapplication of vonoprazan 20 mg/kg markedly reduced these levels compared to the cisplatin-treated rats, while vonoprazan 10 mg/kg significantly decreased BUN and LDH levels (Figure 1).

#### 3.1.2 Influences of vonoprazan on cisplatin-mediated histopathological alterations in the kidney, stomach, duodenal, and jejunal tissues

##### 3.1.2.1 Kidney

**FIGURE 2:**
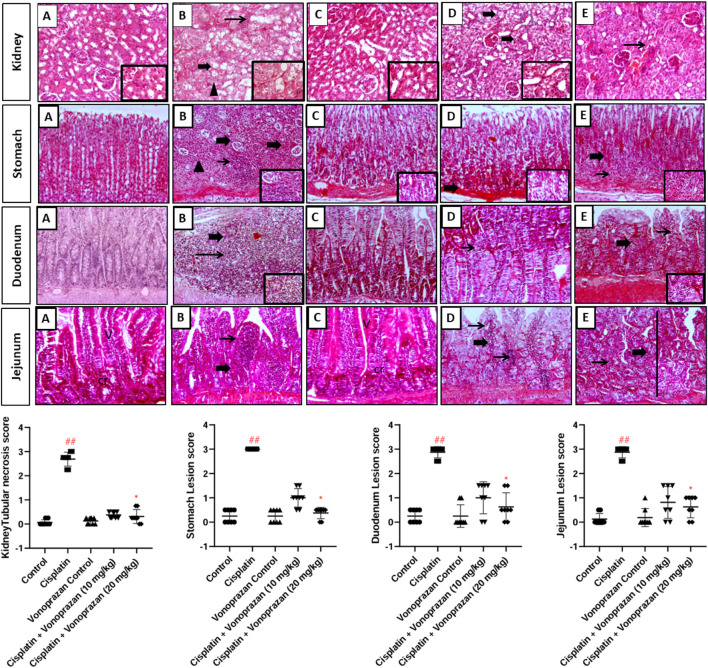
Influences of vonoprazan on cisplatin-mediated histopathological alterations in the kidney, stomach, duodenal, and jejunal tissues based on hematoxylin and eosin staining. Top: Representative photomicrographs of tissues from different treatment groups: **(A)** control, **(B)** cisplatin, **(C)** vonoprazan-only, **(D)** cisplatin + vonoprazan (10 mg/kg), and **(E)** cisplatin + vonoprazan (20 mg/kg) groups. Bottom row: scoring for kidney tubular necrosis, stomach lesions, duodenal lesions, and jejunal lesions. In the stained images, thin arrows indicate fibrosis + inflammation, while thick arrows indicate tubular swelling and necrosis; the arrowheads indicate hyaline degeneration or intraluminal desquamated epithelium (cellular cast), while the circular structures indicate tubular dilation. V: villi, cr: crypt, thin arrow: inflammatory aggregation, line: proliferative crypt, thick arrow: glandular change as dilation, necrosis, or abscess. Image magnification = ×100, inset = ×400. Cisplatin was administered as a single dose (8 mg/kg, i.p.) on the seventh day, while vonoprazan was administered at two dose levels (10 or 20 mg/kg) orally for 10 d. The data are expressed as the median ± interquartile range (IQR) (n = 4) and statistically analyzed using Kruskal–Wallis test, followed by Dunn’s multiple comparison test; ## indicates a highly significant difference over the control group, and * represents a significant difference over the cisplatin group.

As shown in Figure 2, the control and vonoprazan-only groups have normal architectures of the glomeruli and renal tubules. Although the cisplatin group showed diffuse tubular degeneration and necrosis characterized by either tubular vacuolation or shrunken hypereosinophilic epithelial cells with sloughed epithelial cells filling the lumen and forming a granular cast (inset), there was marked tubular desquamation. Alternatively, the cisplatin + vonoprazan (10 mg/kg) group displayed mild tubular degeneration marked by swollen epithelial cells with vacuolated cytoplasm and few tubular dilations (tubular epithelial cell vacuolation); the cisplatin + vonoprazan (20 mg/kg) group revealed mild tubular degeneration with peritubular focal area of inflammatory aggregates admixed with hemorrhage. Figure 2 shows substantially greater scores for renal tubular necrosis in the cisplatin-treated rats than the controls but notably lower values for the two management groups.

##### 3.1.2.2 Stomach

As presented in Figure 2, the control and vonoprazan-only groups reveal normal histological appearance of gastric pits and tubular glands. In contrast, the cisplatin group showed complete loss of gastric mucosa architecture as well as dilation of the gastric glands lined with attenuated epithelium and filled with dead and live neutrophils; in addition, there was marked expansion of the lamina propria and submucosa with fibrosis admixed with multifocal extensive-to-coalescing aggregation of numerous lymphocytes, macrophages, plasma cells, and neutrophils (see inset showing the gland abscess and periglandular aggregation of cellular infiltrates). Although the cisplatin + vonoprazan (10 mg/kg) group displayed minimal glandular epithelial vacuolation, the cisplatin + vonoprazan (20 mg/kg) group showed moderate glandular necrosis with periglandular coalescing aggregation of abundant eosinophils, macrophages, and lymphocytes. Figure 2 indicates considerably greater scores for stomach lesions in the cisplatin-treated rats than the controls but markedly lower values for the two management groups.

##### 3.1.2.3 Duodenum

As demonstrated in Figure 2, the control and vonoprazan-only groups show normal mucosal villi with crypts and submucosal layer, whereas the cisplatin group shows marked mucosal thickening and extensive replacement of the lamina propria with marked aggregation of inflammatory cells (leading to loss of normal villous architecture) and extensive aggregations of lymphoplasmacytic cells mixed with eosinophils surrounding the degenerated crypts. However, the cisplatin + vonoprazan (10 mg/kg) group revealed focal aggregations of mild-to-moderate numbers of inflammatory cells in the lamina propria with aggregation of lymphocytes, plasma cells, macrophages, and fibroblasts, while the cisplatin + vonoprazan (20 mg/kg) group showed villous fusion with multifocal apical villous expansion and fewer numbers of inflammatory cells. Figure 2 indicates that the duodenum lesion score is considerably elevated for the cisplatin-treated rats than the controls but markedly lower for the two management groups.

##### 3.1.2.4 Jejunum

As presented in Figure 2, the normal control and vonoprazan-only groups reveal normal architectures of the mucosal villi and crypts, with a normal muscular layer. Although the cisplatin group showed severe mucosal thickening with transmural expansion of the lamina propria and epithelial degeneration, there was severe aggregation of lymphocytes, plasma cells, and macrophages replacing the lamina propria. The cisplatin + vonoprazan (10 mg/kg) group revealed moderate crypt degeneration represented by dilation of the crypt epithelium and scattered multifocal lamina propria aggregation of the inflammatory cells, along with pericryptal aggregation of lymphocytes, plasma cells, and eosinophils surrounding the swollen vacuolated crypts; the cisplatin + vonoprazan (20 mg/kg) group revealed moderate crypt proliferation with mild crypt vacuolation and inflammation of the lamina propria, in addition to fewer inflammatory cells between the proliferated crypts. Figure 2 indicates that the jejunum lesion score is notably higher in the cisplatin-treated rats than the controls but notably lower in the two management groups.

Following the initial evaluation of the kidney function biomarkers and histopathological examination, we confirmed that the higher dose of vonoprazan (20 mg/kg) was more efficient than the lower dose (10 mg/kg) in attenuating cisplatin-induced tissue injury to some extent. Therefore, subsequent mechanistic studies were performed with only the higher dose of vonoprazan. This approach allowed us to focus on elucidating the underlying mechanisms, which is more feasible when using a single dose, while minimizing the experimental complexity and costs.

#### 3.1.3 Influences of vonoprazan (20 mg/kg) on cisplatin-mediated alterations in the oxidant/antioxidant balance in the kidney, stomach, and intestinal tissues

**FIGURE 3:**
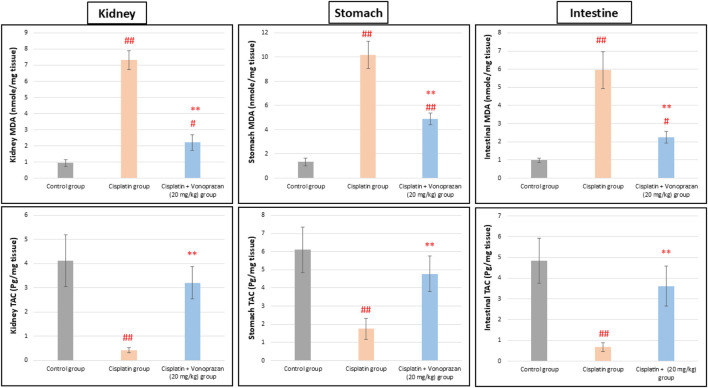
Influences of vonoprazan (20 mg/kg) on cisplatin-mediated alterations in the oxidant/antioxidant balance in the kidney, stomach, and intestinal tissues. The data are presented as the mean ± SD (n = 7). Cisplatin was administered as a single dose (8 mg/kg, i.p.) on the seventh day, while vonoprazan (20 mg/kg) was given orally for 10 d. Statistical evaluations were carried out using one-way ANOVA, followed by the Tukey–Kramer *post hoc* test; # denotes a significant difference over the control group, ## indicates a highly significant difference over the control group, * represents a significant difference over the cisplatin group, and ** indicates a highly significant difference over the cisplatin group. MDA, malondialdehyde; TAC, total antioxidant capacity.

Cisplatin administration significantly increased MDA concentrations in the kidney, stomach, and intestinal tissues while significantly reducing TAC compared to the control group. Vonoprazan (20 mg/kg) pretreatment notably reduced MDA and elevated TAC levels in the kidney, stomach, and intestinal tissues compared to the cisplatin group (Figure 3).

#### 3.1.4 Influences of vonoprazan (20 mg/kg) on cisplatin-mediated alterations in the inflammasome component (NLRP3) and inflammatory signaling mediators (NF-κB and IL-6)

**FIGURE 4:**
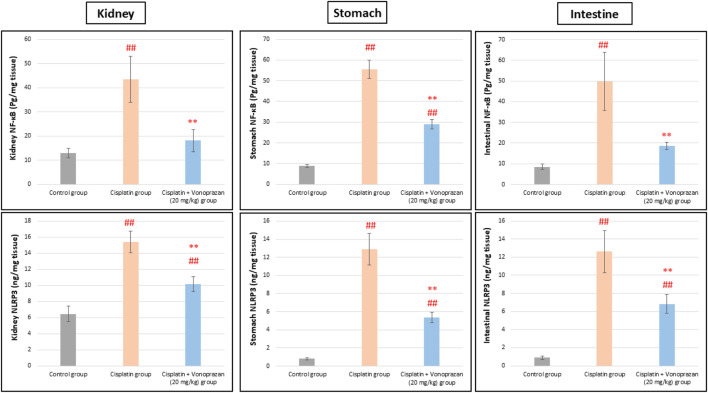
Influences of vonoprazan (20 mg/kg) on cisplatin-mediated alterations in the inflammasome component (NLRP3) and inflammatory signaling mediator (NF-κB). The data are presented as the mean ± SD (n = 7). Cisplatin was administered as a single dose (8 mg/kg, i.p.) on the seventh day, while vonoprazan (20 mg/kg) was given orally for 10 d. Statistical evaluations were carried out using one-way ANOVA, followed by the Tukey–Kramer post hoc test; # denotes a significant difference over the control group, ## indicates a highly significant difference over the control group, * represents a significant difference over the cisplatin group, and ** indicates a highly significant difference over the cisplatin group. NF-κB, nuclear factor kappa B subunit; NLRP3, NOD-, LRR-, and pyrin domain-containing protein 3.

**FIGURE 5:**
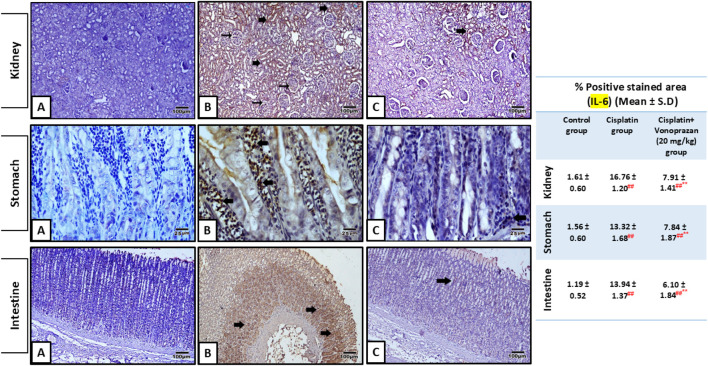
Influences of vonoprazan (20 mg/kg) on cisplatin-mediated alterations in kidney, stomach, and intestinal expression levels of IL-6 using immunohistochemical assay. Kidney sections: **(A)** control group showing negative IL-6 immunostaining of podocytes or renal tubules; **(B)** cisplatin group showing strong IL-6 immunostaining (thin arrows) of podocytes in the glomeruli and tubular cells (thick arrows); **(C)** cisplatin + vonoprazan (20 mg/kg) group showing weak IL-6 immunostaining (thick arrow). Image magnification = ×400. Stomach sections: **(A)** control group showing negative IL-6 immunostaining; **(B)** cisplatin group showing strong IL-6 immunostaining (thick arrows) in the fundic glands; **(C)** cisplatin + vonoprazan (20 mg/kg) group showing weak IL-6 immunostaining (thick arrow) in the fundic glands. Image magnification = ×400. Intestinal sections: **(A)** control group showing negative IL-6 immunostaining; **(B)** cisplatin group showing strong IL-6 immunostaining in the cells of lamina propria of the intestinal villi (thick arrows); **(C)** cisplatin + vonoprazan (20 mg/kg) group showing weak IL-6 immunostaining (thick arrow) in the cells of the lamina propria of the intestinal villi. Image magnification = ×400. The table shows percentage of stained areas in different groups. The data are expressed as the mean ± SD (n = 5). Cisplatin was administered as a single dose (8 mg/kg, i.p.) on the seventh day, while vonoprazan (20 mg/kg) was given orally for 10 d. Statistical analyses were performed using one-way ANOVA, followed by the Tukey–Kramer *post hoc* test; # denotes a significant difference over the control group, ## denotes a highly significant difference over the control group, * indicates a significant difference over the cisplatin group, and ** indicates a highly significant difference over the cisplatin group.

There was significant upregulation in the kidney, stomach, and intestinal NF-κB/NLRP3/IL-6 inflammatory axis upon cisplatin administration compared to the control group. Conversely, vonoprazan co-administration considerably decreased the kidney, stomach, and intestinal levels of NF-κB, NLRP3, and IL-6 compared to the cisplatin group (Figures 4, 5).

#### 3.1.5 Influences of vonoprazan (20 mg/kg) on cisplatin-mediated alterations in the autophagy-lysosomal pathway: LC3B/Beclin-1/SQSTM1 (p62)

**FIGURE 6:**
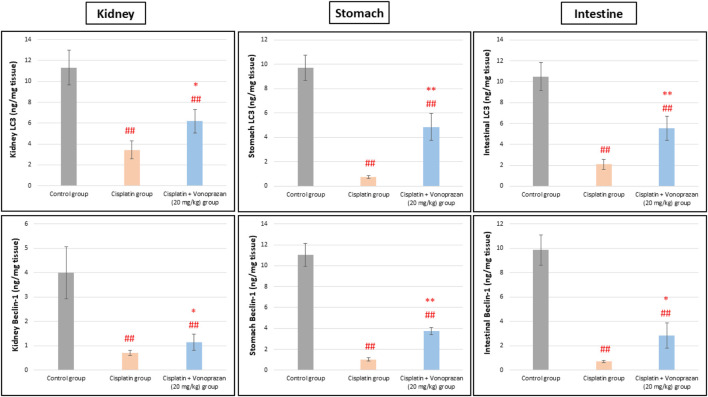
Influences of vonoprazan (20 mg/kg) on cisplatin-mediated alterations in the autophagy-lysosomal pathway. The data are presented as the mean ± SD (n = 7). Cisplatin was administered as a single dose (8 mg/kg, i.p.) on the seventh day, while vonoprazan (20 mg/kg) was given orally for 10 d. Statistical evaluations were carried out using one-way ANOVA, followed by the Tukey–Kramer *post hoc* test; # denotes a significant difference over the control group, ## indicates a highly significant difference over the control group, * represents a significant difference over the cisplatin group, and ** indicates a highly significant difference over the cisplatin group. LC3B, microtubule-associated protein 1A/1B-light chain 3 beta; Beclin-1, coiled-coil moesin-like BCL2-interacting protein.

**FIGURE 7:**
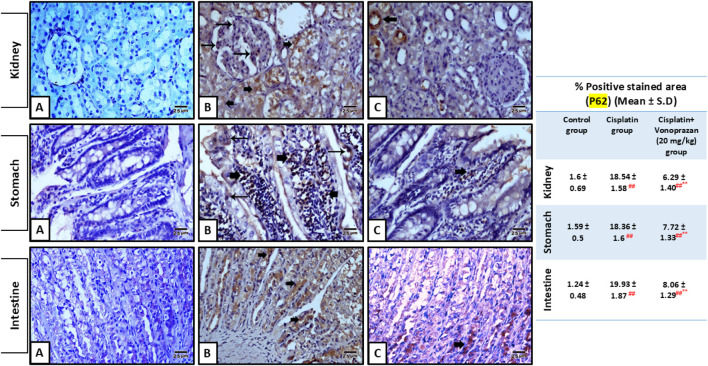
Influences of vonoprazan (20 mg/kg) on cisplatin-mediated alterations in the kidney, stomach, and intestinal expressions of SQSTM1 (p62) based on the immunohistochemical assay. Kidney sections: **(A)** control group showing negative p62 immunostaining in the tubular cells or podocytes; **(B)** cisplatin group showing strong p62 immunostaining (thin arrows) of the podocytes in the glomeruli and tubular cells (thick arrows); **(C)** cisplatin + vonoprazan (20 mg/kg) group showing weak p62 immunostaining (thick arrow). Image magnification = ×400. Stomach sections: **(A)** control group showing negative p62 immunostaining; **(B)** cisplatin group showing strong p62 immunostaining (thick arrows) in the fundic glands; **(C)** cisplatin + vonoprazan (20 mg/kg) group showing weak p62 immunostaining (thick arrow) in the fundic glands. Image magnification = ×400. Intestinal sections: **(A)** control group showing negative p62 immunostaining; **(B)** cisplatin group showing strong p62 immunostaining in the epithelial cells (thin arrows) and in cells of the lamina propria of the intestinal villi (thick arrows); **(C)** cisplatin + vonoprazan (20 mg/kg) group showing weak p62 immunostaining (thick arrow) in cells of the lamina propria of the intestinal villi. Image magnification = ×400. The table shows the percentage of stained areas in different groups. The data are expressed as mean ± SD (n = 5). Cisplatin was administered as a single dose (8 mg/kg, i.p.) on the seventh day, while vonoprazan (20 mg/kg) was given orally for 10 d. Statistical analyses were performed using one-way ANOVA, followed by the Tukey–Kramer *post hoc* test; # indicates a significant difference over the control group, ## indicates a highly significant difference over the control group, * denotes a significant difference over the cisplatin group, and ** denotes a highly significant difference over the cisplatin group.

Cisplatin administration caused an obvious defect in the autophagy process, as evidenced by the significantly decreased levels of LC3B and Beclin-1 along with significant elevations in the expression level of p62 in the kidney, stomach, and intestinal tissues compared to the control group. Contrarily, pre-administration of vonoprazan markedly improved the autophagy process, as indicated by the significant elevations in LC3B and Beclin-1 levels along with profound decreases in the expression level of p62 in the kidney, stomach, and intestinal tissues compared to the cisplatin group (Figures 6, 7).

#### 3.1.6 Influences of vonoprazan (20 mg/kg) on cisplatin-mediated alterations in the early biomarkers of acute kidney injury (KIM-1 and NGAL)

**FIGURE 8:**
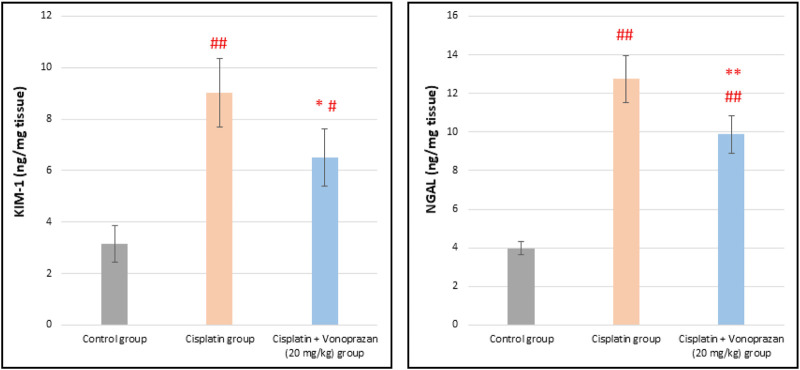
Influences of vonoprazan (20 mg/kg) on cisplatin-mediated alterations in the early biomarkers of acute kidney injury (KIM-1 and NGAL) in the kidney tissue. The data are presented as the mean ± SD (n = 7). Cisplatin was administered as a single dose (8 mg/kg, i.p.) on the seventh day, while vonoprazan (20 mg/kg) was given orally for 10 d. Statistical evaluations were carried out using one-way ANOVA, followed by the Tukey–Kramer *post hoc* test; # denotes a significant difference over the control group, ## indicates a highly significant difference over the control group, * represents a significant difference over the cisplatin group, and ** indicates a highly significant difference over the cisplatin group. KIM-1: kidney injury molecule-1; NGAL: neutrophil gelatinase-associated lipocalin.

As illustrated in Figure 8, the cisplatin group exhibits renal injury that was revealed by the significant elevations in KIM-1 and NGAL levels in the kidney tissue compared to the control group. In contrast, vonoprazan (20 mg/kg) pre-administration significantly reduced these levels compared to the cisplatin group.

#### 3.1.7 Influences of vonoprazan (20 mg/kg) on cisplatin-mediated alterations in gastroprotective mediators (cGMP, PGI2, and 5-HT) and intestinal epithelial and mucosal injury markers (IFABP and TFF3)

**FIGURE 9:**
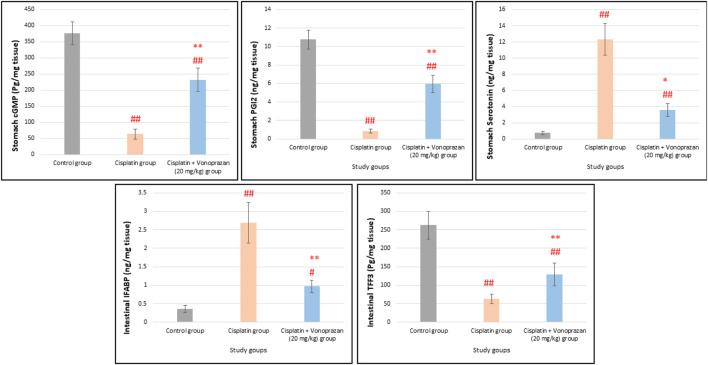
Influences of vonoprazan (20 mg/kg) on cisplatin-mediated alterations in gastrointestinal protective mediators as well as intestinal epithelial and mucosal injury markers. The data are presented as the mean ± SD (n = 7). Cisplatin was administered as a single dose (8 mg/kg, i.p.) on the seventh day, while vonoprazan (20 mg/kg) was given orally for 10 d. Statistical evaluations were carried out using one-way ANOVA, followed by the Tukey–Kramer *post hoc* test; # denotes a significant difference over the control group, ## indicates a highly significant difference over the control group, * represents a significant difference over the cisplatin group, and ** indicates a highly significant difference over the cisplatin group. cGMP, cyclic guanosine monophosphate; PGI2, prostacyclin; 5-HT, serotonin; IFABP, intestinal fatty-acid-binding protein; TFF3, trefoil factor 3.

The cisplatin group showed gastric mucosal damage, as indicated by the significant reductions in gastric levels of cGMP and PGI2 along with significant elevation of serotonin level compared to the control group. Vonoprazan (20 mg/kg) pretreatment notably improved gastric integrity by increasing cGMP and PGI2 levels while decreasing serotonin level compared to the cisplatin group (Figure 9). As demonstrated in Figure 9, cisplatin administration caused marked intestinal epithelial and mucosal injury, as revealed by the significant increase in the intestinal level of IFABP (a marker for intestinal mucosal damage) and significant reduction in TFF3 (mucosal protective factor) compared to the control group. In contrast, vonoprazan (20 mg/kg) pretreatment markedly decreased IFABP level and restored TFF3 level compared to the cisplatin group.

### 3.2 *In vitro* study

#### 3.2.1 MTT analysis

**FIGURE 10:**
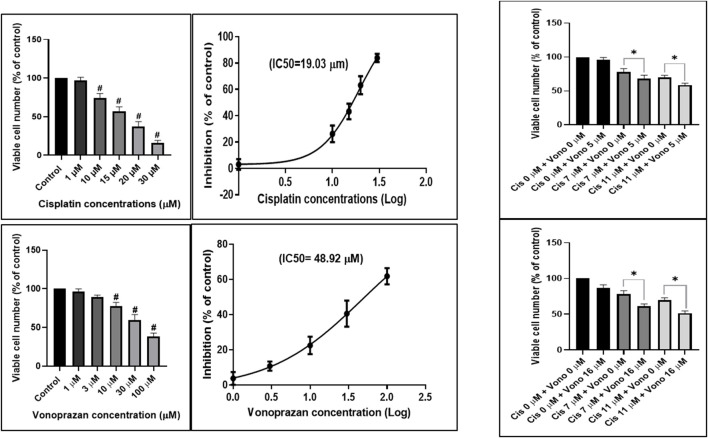
Influences of cisplatin and vonoprazan on human breast cancer (MCF-7) cells after 48 h of exposure; the cytotoxicity was evaluated using the MTT assay in terms of percentage of cell viability by administering increasing concentrations of cisplatin (1, 10, 15, 20, and 30 µM) and vonoprazan (1, 3, 10, 30, and 100 µM). The data are expressed as the mean ± SD. Statistical analyses were performed using one-way ANOVA, followed by Dunnett’s *post hoc* test; # indicates a significant difference over the control group. IC_50_, half of the maximal inhibitory concentration; cis, cisplatin; Vono, vonoprazan.

The MCF-7 cells were exposed to cisplatin or vonoprazan for 48 h to assess their growth-inhibitory effects and determine the cell viability. Cisplatin treatment (1, 10, 15, 20, and 30 µM) showed a dose-dependent decrease in cell proliferation, with an IC_50_ value of 19.03 µM. In contrast, vonoprazan administration (1, 3, 10, 30, and 100 µM) for 48 h notably decreased cell viability (illustrated by greater cytotoxicity), with an IC_50_ value of 48.92 µM (Figure 10). The combination therapy exhibits marked cytotoxicity in the MCF-7 cells treated with vonoprazan (5 and 16 µM) and cisplatin (7 and 11 μM). These cells were initially exposed to vonoprazan (5 or 16 µM) for 48 h and then dosed with cisplatin (7 or 11 μM) for 48 h. The selected cisplatin concentrations maintained cell viability above 50% when used alone. Overall, these findings show that the combined treatment enhances the cytotoxic effects at all concentrations compared to cisplatin as a single agent (Figure 10). The CI results demonstrating the synergistic interactions are summarized in Table 4.

#### 3.2.2 Consequences of combined actions of vonoprazan and cisplatin on p53, BCL2, and BAX protein levels in MCF-7 cells

**FIGURE 11:**
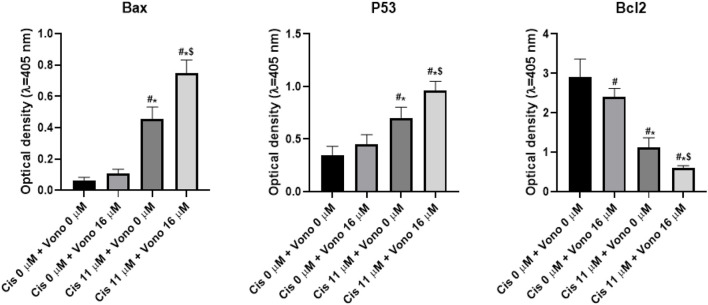
Consequences of combining vonoprazan and cisplatin on the apoptotic effects of cisplatin in MCF-7 cells. The MCF-7 cells were exposed to cisplatin (Cis; 11 µM) and vonoprazan (Vono; 16 µM) individually or in combination for 48 h. The data are expressed as mean ± SD. Statistical analyses were performed using one-way ANOVA followed by the Tukey–Kramer *post hoc* test; the symbols #, *, and $ indicate significant differences over the control (0 µM Cis + 0 µM Vono), vonoprazan-only treatment (0 µM Cis + 16 µM Vono), and cisplatin-only treatment (11 µM Cis + 0 µM Vono) groups, respectively. BCL2, B-cell lymphoma 2; p53, tumor protein p53; BAX, BCL2-associated X protein.

ELISA analysis showed that the combined application of vonoprazan and cisplatin led to substantial elevations in p53 and BAX levels compared to either drug alone or cisplatin alone. Notably, the combination regimen reduced BCL2 level by a greater extent than either agent administered individually (Figure 11). These findings indicate that vonoprazan used in combination with cisplatin could be a promising strategy for enhancing antitumor activity while lowering the drug-induced side effects.

## 4 Discussion

Cisplatin is widely used to treat various malignancies but is limited by its severe nephrotoxic and hepatotoxic side effects (Karasawa and Steyger, 2015). This drug is transported and excreted through proximal tubular transporters, where its accumulation can lead to marked tubular injury, inflammation, and renal toxicity (Perše and Večerić-Haler, 2018). Several compounds have been explored previously for their renoprotective potential against cisplatin-induced nephrotoxicity, although it is uncertain whether such agents could compromise the anticancer effects of cisplatin. Hence, the present study was aimed at evaluating the renoprotective/gastroprotective effects of vonoprazan while preserving or enhancing the antitumor efficacy of cisplatin while minimizing toxicity.

Cisplatin-induced nephrotoxicity entails molecular characteristics with the anticancer mechanisms, but the triggering pathways are not the same. In tumor cells, cisplatin mostly exerts its cytotoxic effect via DNA-platinum adduct formation, which causes DNA crosslinking, replication and transcription inhibition, cell cycle arrest, and apoptosis activation. The rapidly proliferating cancer cells are predominantly vulnerable to this mechanism owing to their high replication rate. In contrast, nephrotoxicity is mainly due to the preferential buildup of cisplatin in the renal proximal tubular epithelial cells via transporters, such as organic cation transporter 2 and copper transporter 1. This accumulation causes extreme reactive oxygen species (ROS) production, mitochondrial dysfunction, ATP depletion, inflammatory pathway (e.g., NF-κB) activation, and pro-inflammatory cytokine (TNF-α and IL-1β) upregulation. Moreover, activation of the apoptotic and necrotic pathways can cause tubular cell injury. Unlike cancer cells, the renal tubular cells are not rapidly dividing; therefore, oxidative stress, inflammation, and mitochondrial damage play more dominant roles in nephrotoxicity than DNA crosslinking alone (Miller et al., 2010; Ozkok and Edelstein, 2014; Pabla and Dong, 2008). Thus, although both anticancer efficacy and nephrotoxicity involve DNA damage and apoptosis, cisplatin-induced kidney injury is largely driven by renal-specific drug accumulation as well as oxidative and inflammatory response activation rather than selective targeting of the proliferating cells.

Cisplatin administration reduced the bodyweight and elevated the kidney/bodyweight ratio, which are attributed to tubular damage that impairs water reabsorption and causes dehydration (Do Amaral et al., 2008; Mi et al., 2018). Owing to its buildup in renal tissue, cisplatin concentration in the kidney surpasses that in the blood (Townsend et al., 2009), leading to nephrotoxicity characterized by elevated sCr and BUN levels (Al Za’abi et al., 2021; Alhoshani et al., 2017; Van Acker et al., 2016). Hence, the elevated sCr and BUN levels in our study along with the results of the histological examination confirmed tubular damage. The histopathological results revealed tubular dilation, epithelial necrosis, and cytolysis in the cortical and medullary regions, consistent with the findings reported by Saifi et al. (2019). As a proximal tubular transmembrane protein, KIM-1 is an early and sensitive indicator of nephrotoxic injury (Khreba et al., 2019; McDuffie et al., 2013). Bonventre (2009) reported that KIM-1 expression is correlated with tubulointerstitial fibrosis and inflammation. Similarly, NGAL serves as a biomarker of acute kidney injury (AKI), and elevated urinary levels of NGAL may be due to enhanced tubular synthesis or impaired reabsorption (Kovacevic et al., 2021). Our findings showed increased KIM-1 and NGAL expressions following cisplatin exposure, thus confirming the development of nephrotoxicity.

Mechanistically, cisplatin-induced AKI involves multifactorial pathways, including oxidative stress, inflammation, and autophagy (Abderrazak et al., 2015; Miller et al., 2010). Cisplatin-induced oxidative stress is a result of excessive ROS generation that exceeds the antioxidant capacity and disturbs redox homeostasis (Kim et al., 2019; Ojha et al., 2016). Consistent with previous reports, cisplatin administration was found to increase MDA and reduce renal TAC levels in our study, confirming oxidative injury and reduced antioxidant enzymes (Abo-Elmaaty et al., 2020). ROS generation has been shown to trigger NLRP3 activation (Abderrazak et al., 2015); NLRP3 is a primary intermediate reactive molecule produced during inflammasome activation that triggers the release of inflammatory cytokines as a form of immune response, thereby intensifying inflammation and worsening tissue damage (He et al., 2016; Kelley et al., 2019). ROS and NLRP3 activation are connected by three main proteins: thioredoxin-interacting protein, NF-κB, and transcription factor nuclear factor erythroid 2-related factor 2 (Nunes et al., 2021). Cisplatin activates NF-κB signaling through IκB degradation and promotes transcription of inflammatory mediators, such as TNF-α, IL-1β, and IL-6 (Abderrazak et al., 2015; Miller et al., 2010), to drive NLRP3 expression. Then, NLRP3 inflammasome activation promotes caspase-1-mediated maturation of IL-1β and IL-18. Thus, IL-6 production is indirectly related to NLRP3 inflammasome activity via NF-κB signaling rather than direct cleaving by caspase-1 through NF-κB activation, which then diminishes NLRP3 inflammasome activity and IL-6 expression (Bauernfeind et al., 2009). Elevated renal NF-κB, NLRP3, and IL-6 levels in our animals treated with cisplatin confirmed the involvement of inflammatory responses because of ROS elevation. Vonoprazan treatment diminished NF-κB activation, thereby reducing NLRP3 inflammasome activation and IL-6 production.

Autophagy and mitophagy may additionally contribute to tubular injury and adaptive responses. Autophagy is responsible for lysosomal degradation of damaged organelles and plays a complex and context-dependent role in cisplatin-induced AKI. While excessive or dysregulated autophagy could cause cell death under certain conditions, accumulating evidence suggests that basal and early autophagic activation in the renal tubular cells may serve as adaptive cytoprotective mechanisms by removing the damaged mitochondria and limiting oxidative stress. Several studies have demonstrated that cisplatin impairs autophagic flux, as evidenced by decreased LC3II and Beclin-1 expressions and p62 accumulation, leading to enhanced tubular injury (Periyasamy-Thandavan et al., 2008; Takahashi et al., 2012). Cisplatin administration reduces LC3II, signifying the prohibition of autophagy. This is in accord with earlier investigational studies (Kahkesh et al., 2025; Sun et al., 2019), whose findings confirmed that cisplatin administration reduces LC3II/LC3I ratio and expression of autophagy-related gene *Atg7*, together with elevated p38 protein level. LC3II and Beclin-1 act as markers that are positively correlated with autophagic activity, while p62 is inversely correlated with autophagy (Karim et al., 2007; Liu et al., 2018; Wei et al., 2018). Throughout our investigation, cisplatin markedly decreased LC3II and Beclin-1 expressions while increasing p62 level, indicating diminished autophagy levels during cisplatin-induced AKI; this effect was reversed by vonoprazan upon co-treatment with cisplatin. Vonoprazan treatment restored the autophagy-related protein expressions, suggesting that its nephroprotective effects may be mediated in part by autophagic flux recovery and cellular adaptive response enhancement rather than autophagy inhibition. Collectively, our findings suggest that vonoprazan exerts a protective effect on the kidneys against cisplatin-induced toxicity. This nephroprotective action is likely mediated by the mitigation of oxidative damage and inflammation as well as restoration of diminished autophagy, which are key mechanisms underlying the adverse effects of cisplatin, thereby preserving renal function and ameliorating the harmful side effects associated with cisplatin treatment. The existing findings highlight the probable supportive effects of vonoprazan in improving the safety of cisplatin-based chemotherapy.

With regard to the GI effects of cisplatin, our findings verified that cisplatin could induce gastroenteric mucosal injury, as evidenced by the pathological alterations to the stomach, duodenum, and jejunum. Our findings are in agreement with extant reports that mucosal injury occurs 3–7 d after cisplatin application (Tazuke et al., 2011). The gastric mucosal cells secrete a mucus-like substance that acts as a protective barrier for the stomach wall, while the parietal cells play crucial roles in maintaining gastric tissue integrity by generating hydrochloric acid to regulate microbial growth within the stomach. In our study, cisplatin administration led to marked decreases in both mucosal and parietal cell populations. Furthermore, our findings are consistent with those of Yamamoto et al. (2013), who reported impaired mucus barrier function following cisplatin administration. However, co-administration of vonoprazan provided substantial protection to all structures against cisplatin-induced damage. Disruption of the structural integrity of the gastric tissue by cisplatin could probably elucidate the noteworthy reduction in dietary utilization and consequent failure of percentage weight alteration (Kim et al., 2023) in the cisplatin group. Nonetheless, such outcomes were improved in the vonoprazan pretreated groups.

The GI tract is the primary source of ROS in the body. Although the epithelial layer affords a protective barrier, ingested substances and pathogens can still trigger inflammation by provoking the epithelium, polymorphonuclear neutrophils, and macrophages to generate inflammatory cytokines and related mediators that can exacerbate oxidative stress. Cancer chemotherapy is frequently associated with adverse toxic reactions, where ROS creation by the chemotherapeutic agents is the primary mechanism underlying toxicity. Elevated lipid peroxidation as well as diminished antioxidant and tissue glutathione levels have been noted during chemotherapy (Conklin, 2004; Khan et al., 2012). Adefisayo et al. (2018) revealed that cisplatin could possibly trigger gastric damage by inducing oxidative stress, increasing the pro-inflammatory cytokine levels, and promoting the infiltration of inflammatory cells in gastric tissue. Our investigation showed a notable protective action of vonoprazan against cisplatin-induced mucosal damage, as evidenced primarily by the modulation of oxidative stress after cisplatin administration markedly elevated MDA levels (indicating excessive lipid peroxidation and oxidative tissue damage) while significantly reducing TAC levels (reflecting depletion of the inherent antioxidant protection system). Sallam et al. (2025) confirmed the antioxidant ability of vonoprazan as a giardicidal agent, and these effects may be linked to the potent anti-inflammatory, anti-apoptotic, and antioxidant activities of vonoprazan. ROS have been implicated in various GI inflammatory disorders, including gastroduodenal inflammation, ulcer formation, and gastric cancer (Phull et al., 1995). Extreme ROS levels have been known to harm the cellular proteins and eventually interrupt GI tract barrier to increase the permeability of the stomach, leading to inflammation in many GI disorders (Rao et al., 1999). Inflammation subsequently plays a crucial role in intestinal epithelial barrier injury; there are medications that help prevent intestinal barrier dysfunction and offer anti-inflammatory protection (Camilleri et al., 2012; Chi et al., 2018). The compromised intestinal epithelial layer integrity also activates inflammatory responses.

Elevated levels of inflammatory mediators in the intestinal epithelial cells can significantly contribute to intestinal inflammation (Cario et al., 2000; Jung et al., 1995). NF-κB act as a potent redox sensor and is activated by oxidative stress (Conklin, 2004); it is triggered by miscellaneous motivators that can possibly increase host risk, leading to the stimulation of inflammatory and immunological reactions (Aboubakr et al., 2022). NLRP3 inflammasomes are implicated in the control of inflammation induced by several non-infectious causes, such as lipid buildup, oxidative stress, and intestinal barrier injury (Kelley et al., 2019; Zhen and Zhang, 2019); these inflammasomes stimulate caspase-1 and enable discharge of pro-inflammatory cytokines IL-1β/IL-18/TNF-α (Kelley et al., 2019). Here, the inflammatory reactions prompted by the oxidative injury caused by cisplatin manifested as notable alterations in the levels of NF-κB, NLRP3, and IL-6, which are crucial moderators of the inflammatory cascade that cause mucosal interruption. Co-treatment with vonoprazan effectively downregulated these pro-inflammatory markers, thereby inhibiting NF-κB/NLRP3/IL-6 and reducing cisplatin-induced inflammation.

At the cellular level, autophagy dysregulation encouraged by cisplatin was evidenced by enhanced p62 buildup and lowered expressions of LC3 and Beclin-1, suggesting diminished autophagic flux. Vonoprazan meaningfully ameliorated the p62, LC3, and Beclin-1 disturbances induced by cisplatin to recover autophagic clearance and improve cellular homeostasis. We assessed the IFABP and TFF3 levels to evaluate the effects of cisplatin on intestinal injury and probable protection offered by vonoprazan. IFABP is a cytosolic protein that acts as an essential factor in the cellular uptake and is expressed by the enterocytes in the small intestine; it is discharged when the GI mucosal integrity is compromised and there is mucosal damage. Extensive research efforts have shown that IFABP is a valuable indicator of acute intestinal ischemia and inflammatory bowel impairment (Linsalata et al., 2020; Pelsers et al., 2005). TFF3 is a glycoprotein secreted by the goblet cells that has vital actions in protecting the mucosal lining. Research has shown that TFF3 controls various GI disorders, such as ulcerative colitis and Crohn’s disease (Belle et al., 2019). Our investigation revealed that the observed intestinal mucosal damage was associated with elevated IFABP and diminished TFF3 levels upon cisplatin administration; these effects were reversed upon vonoprazan co-administration, indicating mucosal repair and epithelial regeneration.

NO has gastroprotective effects and is involved in numerous intracellular signaling pathways, including the elevation of cGMP levels (Medeiros et al., 2008). cGMP is known to trigger various cGMP-dependent protein kinases that activate ATP-sensitive potassium channels, which are abundantly expressed in the smooth muscle cells of the guinea pig stomach (Sim et al., 2002); blocking these channels has been shown to negate the observed gastroprotective action (Díaz-Triste et al., 2014). The arachidonic acid metabolites PGI2 also participates in gastric mucosal protection, and its role in protecting the gastric mucosa from diverse injurious factors is well recognized. PGI2 is also known to impede leukocyte stimulation (Eisenhut et al., 1993; Isobe et al., 1999). Serotonin secreted by the intestinal enterochromaffin cells motivates the 5-HT_3_ receptors on the vagal afferent fibers and enteric nerves; this relaxes the stomach and mainly the fundus (Martín-Ruíz et al., 2019) by motivating the vago-vagal reflex, leading to 5-HT_3_ antagonist-sensitive delayed gastric emptying and distension (Martín-Ruíz et al., 2019; Vera et al., 2014) as well as gastric retention of food (Aggarwal et al., 1994; Malik et al., 2007) and probably gas (Malik et al., 2007). In our work, cisplatin caused significant alterations to the gastric cGMP and PGI2 levels, further promoting mucosal injury with elevated 5-HT level that confirmed altered GI motility and local irritation. Our findings are in accordance with the results of Nakajima et al. (1996), who proved that cisplatin influences liberation of 5-HT from the enterochromaffin cells in the mucosa, thereby causing emesis through motivation of the 5-HT_3_ receptors. In our study, vonoprazan was found to normalize cGMP, PGI2, and serotonin levels, thereby contributing to the stabilization of GI motility and reduction of local irritation.

Interestingly, in our *in vitro* study, co-treatment with vonoprazan and cisplatin resulted in a marked reduction in MCF-7 cell viability compared to single-drug treatments. Analysis of the drug interactions using the CI metric revealed a synergistic effect, confirming that the combined regimen enhances cytotoxic activity beyond those of the individual agents. Dysfunction of the mitochondrial apoptotic pathways is closely linked to tumor development and resistance of various cancer cells to cytotoxic drugs (Czabotar et al., 2014; Sharifat et al., 2017). As a key tumor suppressor, p53 plays a crucial role in regulating apoptosis. Our ELISA results showed that treatment with either vonoprazan or cisplatin increased p53 protein levels; however, co-treatment with both drugs significantly increased p53 level compared to each treatment alone. Apoptosis induction involves multiple effectors acting via intrinsic or extrinsic pathways. The BCL2 family of proteins comprises both pro-apoptotic and anti-apoptotic members and is a vital controller of the p53-mediated apoptotic pathway. The activation of p53 implies a high BAX (pro-apoptotic) to BCL2 (anti-apoptotic) ratio, which is crucial for augmenting cellular sensitivity to apoptosis. This process includes mitochondrial outer membrane permeabilization, discharge of cytochrome c into the cytoplasm, and subsequent caspase initiation, ending in cell death (Czabotar et al., 2014). Anticancer treatments frequently lead to increased BAX/BCL2 ratio by either decreasing BCL2 expression or increasing BAX level (Fattah et al., 2021; Sharifi et al., 2015).

The protective and modulatory actions observed with vonoprazan in our work may be linked to those conveyed by proton pump inhibitors (PPIs), specifically omeprazole. PPIs are widely used to prevent or reduce chemotherapy-induced gastric irritation by inhibiting gastric H^+^/K^+^-ATPase following acid activation. Extant studies indicate that omeprazole may exert additional antioxidant and anti-inflammatory actions to potentially diminish oxidative-stress-induced tissue injury, including renal damage (Luciani et al., 2004; Spugnini et al., 2010). Nevertheless, there are vital pharmacological variations between PPIs and vonoprazan. In contrast to omeprazole that needs acidic activation and displays delayed onset of action, vonoprazan is a P-CAB that produces rapid, reversible, and more sustained inhibition of gastric acid secretion independent of acid activation. This results in more stable intragastric pH control, which provides superior mucosal protection during acute cisplatin exposure (Rawla et al., 2018). Earlier studies have also shown that PPIs may affect cisplatin sensitivity by modulating the acidity of the tumor microenvironment and regulating the intracellular pH level (Luciani et al., 2004; De Milito and Fais, 2005; Taylor et al., 2015). The related mechanisms could contribute to the increased anticancer activity of vonoprazan in the combination therapy. However, in contrast to conventional PPIs, vonoprazan displays stronger acid suppression and a favorable pharmacokinetic profile, which could explain the magnitude of protective and synergistic effects observed in this study.

In conclusion, cisplatin causes AKI via interlinked mechanisms of oxidative stress, inflammation, and autophagy. To the best of our knowledge, extant results have established that vonoprazan efficiently moderates cisplatin-induced renal damage by reducing oxidative imbalance, lower inflammatory responses, and impaired autophagic initiation. In terms of the GI disturbances, vonoprazan offers defensive outcomes against cisplatin-induced mucosal injury through multifactorial mechanisms involving potent antioxidant actions, restriction of NF-κB/NLRP3-mediated inflammation, repair of autophagic balance, and encouragement of mucosal healing to improve FTT3/IFABP, cGMP/PGI2, and 5-HT levels. These highlight the potential of vonoprazan as a promising therapeutic agent for preventing or ameliorating the deleterious GI effects of cisplatin. Further, the preliminary findings of this study suggest that combining vonoprazan with cisplatin may be an effective strategy for treating breast cancer. Lastly, p53/BAX/BCL2 apoptotic signaling appears to be a key mediator of the synergistic interactions between these two drugs.

## 5 Limitations

Regardless of the promising findings of the present study, various limitations should be recognized. First, the cytotoxicity assay was conducted using the MCF-7 breast cancer cell line; expanding the research by including various cancer cell lines would strengthen the generalizability of the results and determine whether the synergistic effects of vonoprazan and cisplatin noted herein are cell-type specific. Second, further mechanistic studies must be conducted in tumor models *in vivo*, including functional assays, genetic or pharmacological inhibition studies, and time-course measurements, to directly assess the combined drug effects on antitumor efficacy and organ protection; these are important for decisively ascertaining the relationships and expansively validating the molecular effects noted herein while improving the robustness and translational relevance of the findings. Finally, we clarify that although the acute model is suitable for studying the mechanistic aspects of cisplatin-induced organ injury and evaluating protective interventions, such as vonoprazan, extrapolation to chronic and multicycle chemotherapy scenarios should be approached with caution. Thus, future studies employing repeated-dose or clinically relevant cisplatin regimens are warranted to fully assess the therapeutic efficacy and translational relevance of the present findings.

## Data Availability

The original contributions presented in the study are included in the article/supplementary material; further inquiries can be directed to the corresponding author.
